# Correlation of retinal alterations with vascular structure of macular neovascularisation in swept-source optical coherence tomography angiography in age-related macular degeneration

**DOI:** 10.1007/s10792-021-02149-6

**Published:** 2022-01-13

**Authors:** Henrik Faatz, Kai Rothaus, Martin Ziegler, Marius Book, Georg Spital, Britta Heimes-Bussmann, Daniel Pauleikhoff, Albrecht Lommatzsch

**Affiliations:** 1 Department of Ophthalmology, St. Franziskus Hospital, Münster, Germany; 2 Achim Wessing Institute for Imaging in Ophthalmology, University Hospital Duisburg–Essen, Essen, Germany; 3 Department of Ophthalmology, University of Duisburg–Essen, Essen, Germany

**Keywords:** MNV-morphology, Imaging, Age-related macular degeneration, OCT-angiography, Choroidal neovascularization

## Abstract

**Purpose:**

The aim of this study was to find out whether the vascular architecture of untreated macular neovascularisations (MNV) in neovascular age-related macular degeneration (nAMD) as visualised with optic coherence tomography angiography (OCTA) is associated with functional and known morphological alterations of the retina in optic coherence tomography (SD-OCT).

**Methods:**

The study design was retrospective with consecutive patient inclusion. In 107 patients with newly diagnosed nAMD, MNV were detected by means of OCTA and automated quantitative vascular analysis was performed. The MNV characteristics measured were area, flow density, total vascular length (sumL), density of vascular nodes (numN), fractal dimension (FD) and average vascular width (avgW). These parameters were assessed for associations with vision (BCVA), central retinal thickness (CRT), fluid distribution, the elevation of any pigment epithelial detachment (PED), the occurrence of subretinal haemorrhage and atrophy.

**Results:**

BCVA was significantly worse with greater MNV area and sumL. Fluid distribution differed significantly in relation to area (*p* < 0.005), sumL (*p* < 0.005) and FD (*p* = 0.001). Greater PED height was significantly associated with higher numN (*p* < 0.05) and lower avgW (*p* < 0.05). Atrophy was present significantly more often in MNV with larger area (*p* < 0.05), higher sumL (*p* < 0.05) and higher flow density (*p* = 0.002). None of the MNV parameters had a significant association with CRT or the occurrence of haemorrhage.

**Conclusion:**

OCTA is not restricted to evaluation of secondary changes but offers the opportunity to analyse the vascular structure of MNV in detail. Differences in vascular morphology are associated with certain secondary changes in retinal morphology. There are thus grounds for optimism that further research may identify and classify OCTA-based markers to permit more individualised treatment of nAMD.

## Introduction

Age-related macular degeneration (AMD) is a potentially vision-impairing disease that represents the leading cause of blindness in the industrialised nations. The prevalence of AMD is rising year by year as life expectancy increases [1]. Indirect stereoscopic ophthalmoscopy permits clinical detection of typical changes affecting the retinal pigment epithelium (RPE), fluid, drusen, haemorrhages, RPE detachment (PED), RPE atrophy or fibrosis, depending on the stage of the AMD. The severity of these changes is extremely heterogeneous both at the time of diagnosis and during the course of the disease, so the treatment required varies widely from case to case, as does the visual impairment. Combined fluorescein angiography (FA) and optic coherence tomography (OCT) is the gold standard for diagnosis of neovascular AMD (nAMD), in which macular neovascularisation (MNV) with subretinal and/or intraretinal exudation occurs. Hyperfluorescent or hypofluorescent distribution of intravenously administered fluorescein reveals vascular pathologies of the retinal and choroid vessels and the presence of subretinal and/or intraretinal fluid can be differentiated [2]. Different types of MNV in nAMD are distinguished according to the findings on FA and OCT [3]. Precise characterisation of vascular morphology is not possible due to the leakage from the MNV. Indocyanine green angiography (ICGA) is often helpful in cases of unclear MNV type.

OCT angiography (OCTA) is a non-invasive imaging modality that offers new insights into the physiological and pathological perfusion of the retinal and choroid by virtue of detection of the movement of red blood cells. OCTA is highly sensitive for detection of MNV [4]. Furthermore, its automated image processing software enables determination of the detailed internal structure and vascular architecture of MNV [5, 6]. The aim of this study was to distinguish different types of MNV vascular architecture and correlate them with other known morphological and functional parameters. The formation and alteration of MNV causes the subsequent morphological changes in Bruch’s membrane, the RPE and the retina, so there are grounds for hope that characteristic features of MNV may serve as biomarkers of disease course and treatment success, thus permitting more individualised therapy.

## Methods

The study was carried out in adherence to the tenets of the Declaration of Helsinki and was approved by the ethics committee of Westphalia–Lippe Medical Association and the University of Münster. Data acquisition was retrospective with consecutive patient inclusion. In all patients, nAMD was first diagnosed by means of FA and SD-OCT (Spectralis© HRA + OCT, Heidelberg Engineering, Heidelberg, Germany) together with clinical examination. The diagnosis was verified by two masked graders (HF, MB) at the reading centre M^3^–Macula Monitor Münster. In case of disagreement between the graders, a senior grader (MZ) made the determination. Data on best corrected visual acuity (BCVA) and the occurrence of retinal haemorrhage were sourced from the medical records. The FA and SD-OCT images were analysed with regard to central retinal thickness (CRT), fluid distribution (intraretinal/subretinal), atrophy and PED.

Moreover, all patients underwent OCTA with the swept-source OCTA PLEX® Elite 9000 (Carl Zeiss Meditec, Dublin, California, USA), working at a wavelength of ~ 1060 nm and 100,000 A-scans/s in a 6 × 6-mm image, records two consecutive sequences of B-scans with 500 A-scans. Automatic suppression of artefacts was employed in order to improve the visualisation of MNV [7]. Patients with inadequate image quality (quality score < 7) and those with retinal pathologies other than nAMD were excluded.

We used the ORCC segment (0 µm from the outer plexiform layer to 49 µm below Bruch’s membrane) for classification. Because morphological changes in the retina frequently lead to incorrect segmentation, all B-scans showing MNV were checked and, whenever necessary, the segmentation lines corrected manually in the scan.

**Fig. 1:**
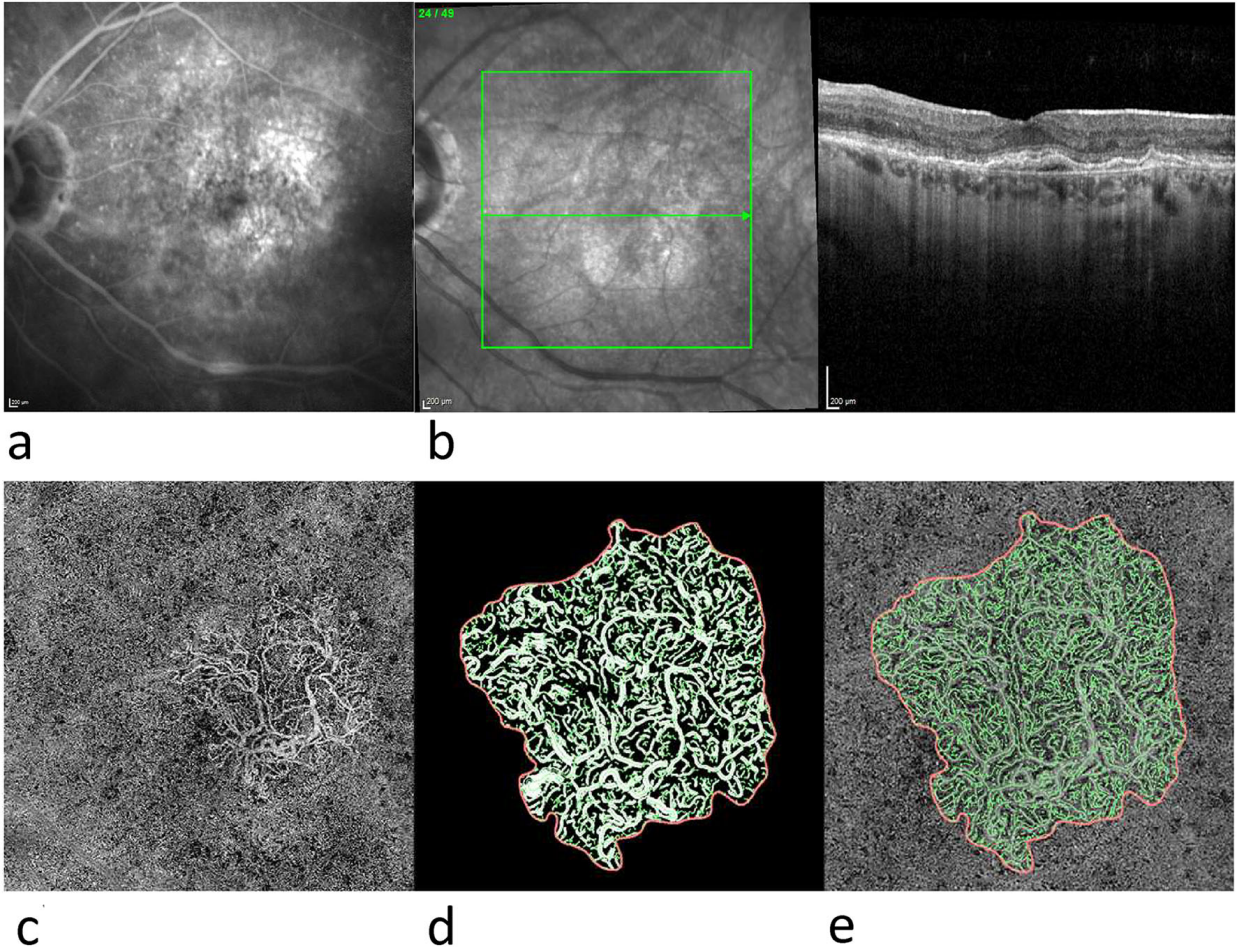
Overview of type 1 MNV: **a** FA, **b** SD-OCT, **c** en-face OCTA of the ORCC slab; **d**, **e** enlarged views of binarised (**d**) and skeletonised (**e**) MNV

**Fig. 2:**
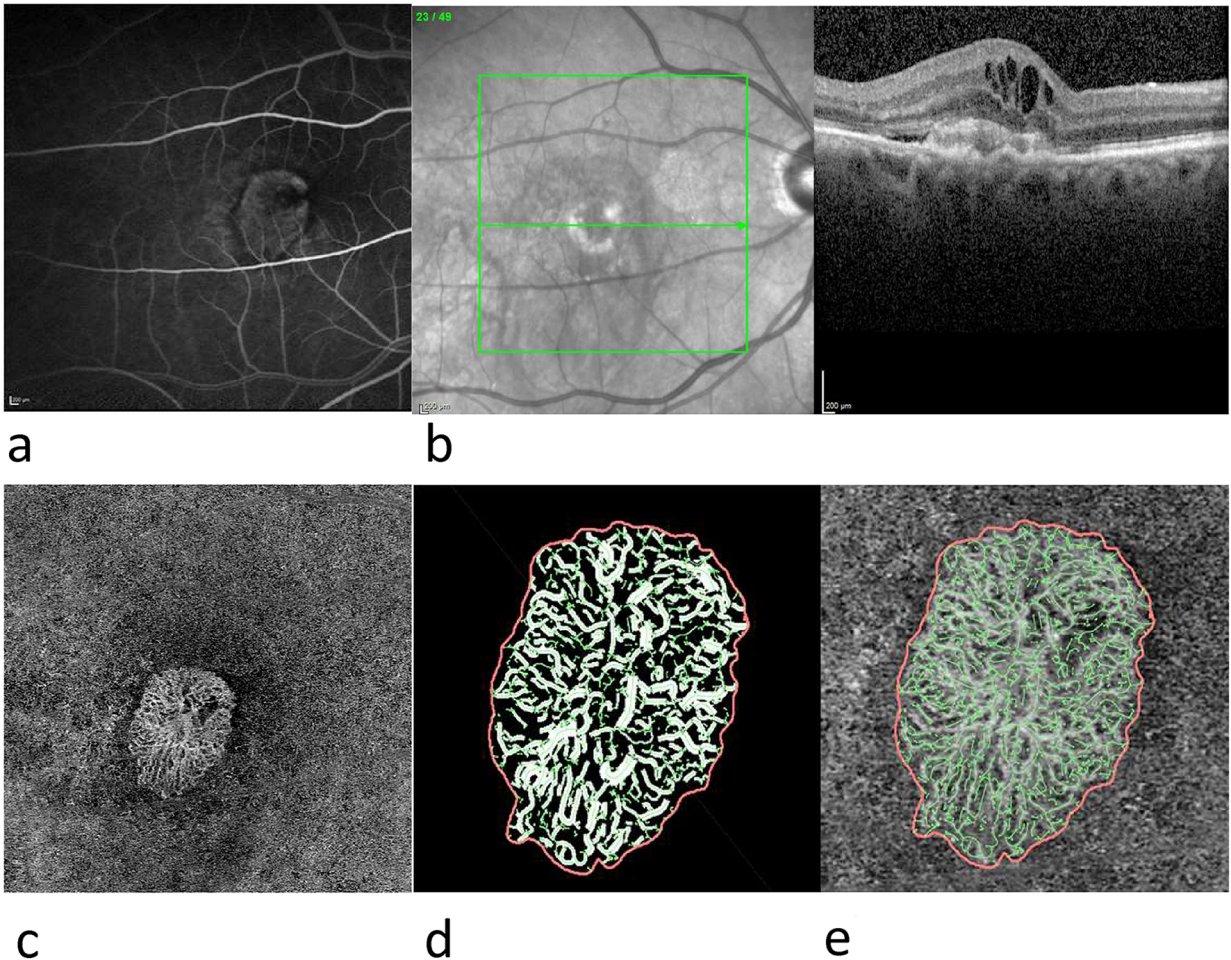
Overview of type 2 MNV: **a** FA, **b** SD-OCT, **c** en-face OCTA of the ORCC slab; **d**, **e** enlarged views of binarised (**d**) and skeletonised (**e**) MNV

In the exported en-face OCTA images we demarcated the MNV with the aid of the program Fiji (National Institute of Mental Health, Bethesda, MD, USA) and isolated them from the remainder of the image for further analysis. The vascular network of each was extracted using MatLab (Mathworks, Version R2014b). On the basis of a multiscale calculation of the gradient field in the en-face OCTA image, the vascular network was skeletonised. This process detects both very thin and thick vascular segments as unbroken midlines. The vascular width was then determined for each vascular segment, so that skeletonisation was accompanied by calculation of vascular network segmentation. The individual vascular segments form the edges of the vascular graphs and the branchings are the nodes. The following six parameters were chosen for morphological characterisation of the MNV: area, flow, fractal dimension (FD), total vascular length (sumL), density of vascular nodes (numN) and average vascular width (avgW). Examples of MNV as visualised on FA, SD-OCT and OCTA scans, together with the binarised and skeletonised MNV, are shown in Figs. 1 and 2.

We compared each of these six vascular parameters with the functional parameter BCVA and with the following morphological parameters of the retina: CRT, IRF/SRF, PED, haemorrhage and presence of an atrophy. The aim was to investigate whether the vascular architecture of MNV has an influence on BCVA or morphological changes in the retina.

Statistical analyses were carried out using R® (Version × 64, 4.0.2) [8]. The level of significance was set at 5%. The presence of normal distribution was verified with the Shapiro–Wilk test, the presence of variance homogeneity with a Levene test. Paired group comparisons of the location parameter were performed using the t-test, providing the preconditions were fulfilled. Alternatively, a corresponding nonparametric test (Wilcoxon rank-sum test or signed-rank test) was used.

In multiple group comparisons an analysis of variance (ANOVA) was carried out, or alternatively a Kruskal–Wallis test. For the post hoc analyses, either the Tukey test (following an ANOVA) or the Dunn test was used, with Bonferroni correction of the *p* values. A correlation test was performed using the Pearson method in the presence of multi-normal distribution, otherwise using the Spearman method.

## Results

We evaluated 169 eyes of 158 patients with MNV in nAMD. After application of the exclusion criteria, 107 eyes remained for analysis. In 17 eyes, no MNV could be discerned despite good image quality. The sensitivity for detection of MNV in our cohort was therefore 84.1%. A further 45 eyes were excluded either because of reduced image quality or due to the presence of another macular disease. The patients’ mean age was 78.1 ± 6.9 years, and their mean BCVA was 0.59 ± 0.33 LogMAR. On the basis of the findings of FA and SD-OCT, 51 of the 107 eyes had type 1 MNV, 33 had type 2, and 23 had type 3.

Table 1 shows the *p* values of the correlations between the OCTA-based vascular parameters (area, FD, numN, Flow, sumL, avgW) and BCVA and the morphological vascular parameters (CRT, IRF/SRF, hemorrhage, PED, atrophy).

**Table 1:** Overview of the correlations (*p* values) of the vascular parameters (area = area of MNV in mm^2^, FD = fractal dimension, numN = number of vascular nodes/mm^2^, sumL = total vessel length, avgW = average vessel width) with BCVA (LogMAR) and retinal morphology (CRT = central retinal thickness, PED = pigment epithelial detachment)

MNV parameter	BCVA	CRT	IRF/SRF	PED	Hemorrhage	Atrophy
area	0,036	0,407	0,004	0,408	0,337	0,017
FD	0,085	0,611	0,001	0,359	0,113	0,068
numN	0,473	0,976	0,384	0,016	0,275	0,208
Flow	0,93	0,341	0,772	0,155	0,955	0,002
sumL	0,05	0,445	0,004	0,407	0,234	0,033
avgW	0,307	0,871	0,774	0,017	0,494	0,068

Table 1: *p* values of the correlations between vascular parameters of MNV with BCVA and retinal morphology.

Vision at the time of diagnosis of nAMD was significantly poorer (*p* < 0.05) in eyes with MNV covering a larger area, although the linear correlation of the parameters was only weak (rho = 0.22). Another negative association was with the sumL of the MNV, which was significantly greater in eyes with worse vision (*p* < 0.05). Again, the linear correlation was weak (rho = 0.21).

Our study found no significant associations between any individual vascular parameter of the MNV and CRT.

**Fig. 3:**
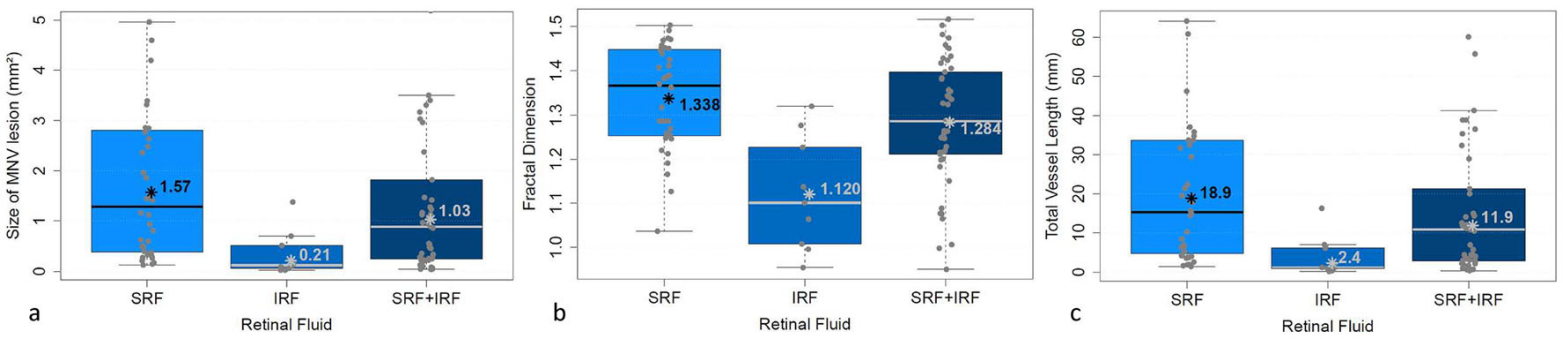
Presence of subretinal fluid (SRF), intraretinal fluid (IRF) or SRF + IRF in relation to the MNV parameters area, FD and sumL. * = mean value, bar = median

The MNV area was significantly larger if only subretinal fluid (SRF) was present than if only intraretinal fluid (IRF) was found (*p* < 0.005), and also significantly larger in the presence of both SRF and IRF than with IRF alone (*p* < 0.05; Fig. 3a).

The same was true for FD: it was significantly greater with SRF than with IRF (*p* < 0.0005) and also significantly greater in the presence of both SRF and IRF than with IRF alone (*p* = 0.01; Fig. 3b).

The parameter sumL, too, was higher both for SRF alone (*p* < 0.005) and for SRF + IRF (*p* < 0.05) than for IRF alone (Fig. 3c).

**Fig. 4:**
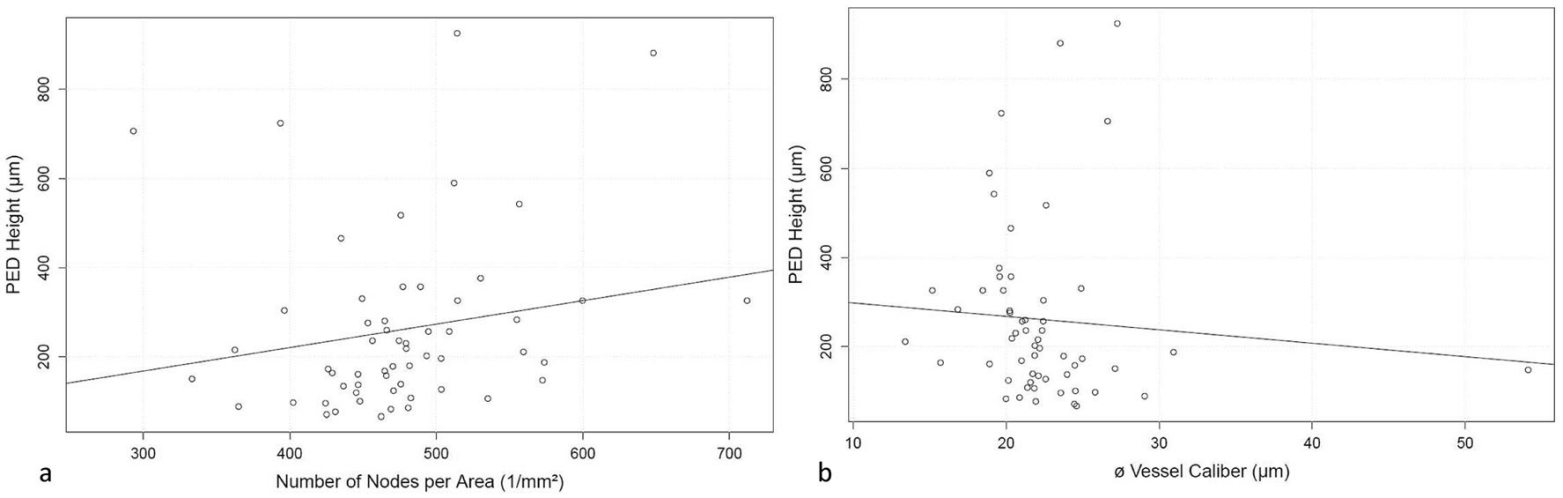
Association of PED height with the vascular parameters **a** numN and **b** avgW

Analysis of PED height revealed a significant positive association with the numN of the MNV (*p* < 0.02), although the parameters showed only a weak linear correlation (rho = 0.32) (Fig. 4a), and a negative association with avgW (*p* < 0.02), again a weak linear correlation (rho = 0.32) (Fig. 4b).

With regard to the occurrence of subretinal haemorrhage, we found no associations or significant differences in connection with any of the vascular configurations.

**Fig. 5:**
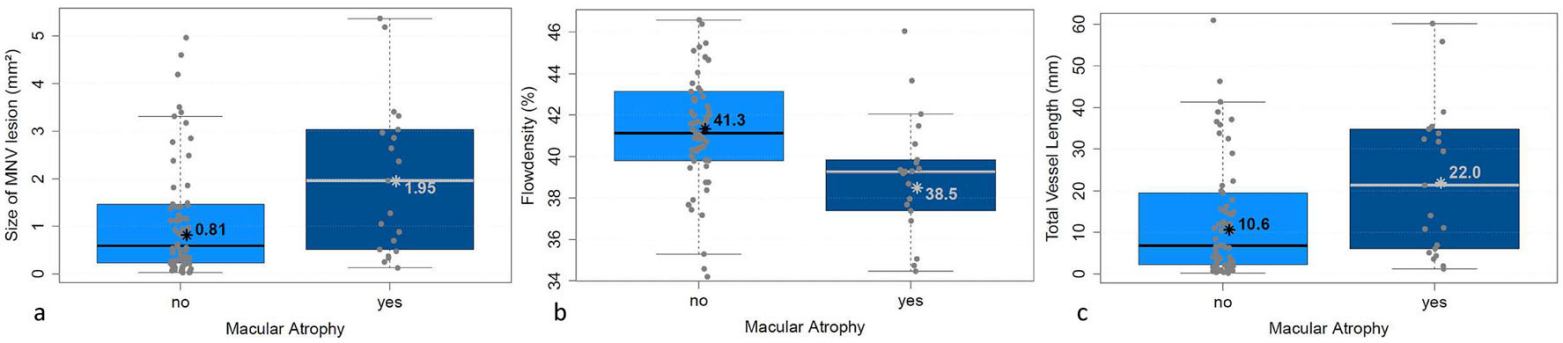
Occurrence of atrophy in relation to **a** area of MNV, **b** flow density of MNV and **c** sumL of MNV. * = mean value, bar = median

Atrophy of the RPE at the time of nAMD diagnosis was found significantly more often in eyes with greater MNV area (*p* < 0.05, Fig. 5a) and significantly less often in eyes with higher flow (*p* = 0.002, Abb. 5b). Another significant way in which the vascular configuration influences the presence of atrophy is that the latter is less often present at the time of nAMD diagnosis when sumL is low (*p* = 0.03, Fig. 5c).

## Discussion

The Consensus Nomenclature for Reporting Neovascular Age-Related Macular Degeneration Study Group emphasises the importance of OCTA as a complementary imaging procedure enabling detailed categorisation of nAMD [3]. The current classification considers the subjective appearance of an MNV and its location with respect to the various segments, but does not take account of the detailed vascular architecture. Nevertheless, a large number of studies have portrayed the vascular morphology of MNV. One study on assessment of MNV activity describes the vascular morphology in the active stage as a dense network of many small branching vessels and capillaries, and after a long period of anti-VEGF treatment as a loose network with larger vessels and fewer branchings [9]. Spaide showed by means of OCTA that the capillaries of MNV decrease in number during anti-VEGF treatment, while the prominent afferent vessels show few or no changes [10]. An earlier study of ours also demonstrated that quantifiable vascular description of MNV is possible and that these structural vascular parameters may change significantly during anti-VEGF treatment [6, 11]. Another parameter of MNV vascular structure is FD; which may also be reduced by anti-VEGF treatment [6, 12]. Moreover, we showed in an earlier study that quantitative analyses are also possible with regard to the total vascular length and number of vascular segments of an MNV, with these parameters showing significant differences between active and inactive disease [6]. However, no clear biomarkers of MNV vascular structure have yet been established that could confirm MNV activity and thus indicate anti-VEGF treatment. It would be desirable to identify biomarkers to prompt initiation of treatment before the occurrence of exudation, in order better to preserve retinal integrity and visual acuity. To this end, it is important to carry out studies investigating MNV vascular morphology and retinal morphology as well as the typical clinical findings of nAMD, such as haemorrhages.

In our study the patients with a larger MNV area and greater total vessel length had poorer vision. This can be explained by the associated more extensive damage to the RPE and the photoreceptors. Other studies have shown that MNV size is also a prognostic factor for the course of vision during anti-VEGF treatment, with an initially large MNV being associated with poorer vision [13–15]. There is an association between the area of the MNV and the total vessel length in our study population. It is obvious that these parameters behave similarly, but it is also possible that there are differences, e.g. between large mature MNV and small MNV with many capillaries. This is probably not reflected in our evaluation, as only active and untreated MNV were examined.

In our study, MNV area, FD, and total vessel length exerted a significant influence on the distribution of subretinal and intraretinal fluid. The larger the MNV (area, sumL) and the more complex the vascular morphology (FD), the more frequently SRF was present, either alone or in combination with IRF. Other studies had already demonstrated that the presence of SRF is a predictive factor for treatment response and the evolution of visual acuity [16, 17]. In the FLUID study, it was demonstrated that central and maximum 200 µm SRF can be tolerated and resulting in the same BCVA and fewer injections after 24 month [18]. The CATT study showed that patients with central SRF were associated with better visual acuity than those without SRF even after 2 years [19]. However, other studies have different results: Grechenig et al. showed using artificial intelligence that tolerating SRF volumes in the central 1 and 6 mm is associated with an increase in fluid volume and subsequently with BCVA loss at the next visit [20]. Simander et al. did not observe an effect of baseline SRF in nAMD on visual recovery [21]. The occurrence of SRF is not only dependent on MNV, it may also be related to changes in the RPE and bruch membrane. The distribution and response of SRF to anti-VEGF therapy seem to be dependent on several factors and so far experience is not sufficient to fully understand it. As fluid is only a secondary effect of MNV, it is important to investigate the vascular morphology of MNV and find differences or similarities.

A PED arises when the RPE is separated from Bruch’s membrane by an accumulation of fluid. This can be observed in various diseases and occurs in 63–80% of eyes with nAMD [22, 23]. We did not distinguish among vascularised, serous and fibrovascular PED. Two of the parameters we analysed were correlated with PED elevation: a high numN and lower avgW were associated with a more elevated PED. This association may be explained by the fact that the surface of small vessels and capillaries is not covered by pericytes, so that MNV rich in such vessels exhibit higher exudation. However, we were not able to demonstrate any significant association of vessel width with the occurrence of SRF and IRF, which points to the influence of other pathological changes, e.g. in the RPE and Bruch’s membrane, on the complex development of PED [24].

If a patient with nAMD develops atrophy of the photoreceptors, the RPE and the choriocapillaris, the result is irreversible visual impairment. We found that the presence of atrophy was associated with larger MNV area and greater vessel length. This connection is corroborated by the association of both of these parameters with poorer vision. Low flow density in MNV was also correlated with the occurrence of atrophy in our study. This may mean that low flow indicates a longstanding MNV, which again is associated with atrophy of the RPE. We ascertained in a previous study that higher flow density is linked with a more favourable visual outcome [15].

The CRT as visualised on SD-OCT plays a major role in assessing whether nAMD requires treatment. For this reason, we expected to find associations between MNV vascular morphology and retinal thickness, but no such link emerged for any of the parameters we analysed. The explanation may be found in the wide variety of morphological structures (IRF, SRF, fibrosis) that determine CRT. This is in accordance with the data of Told et al., who also found no association between the CRT and the area, sumL or numN of MNV. They did, however, show a significant correlation of these parameters with parafoveal and perifoveal retinal thickness [25]. One possible reason for this may be that the two-dimensional measurement of CRT does not adequately capture the three-dimensional MNV. Another explanation is that any increase in retinal thickness due to an MNV also depends on whether the expanding MNV displaces or infiltrates the retinal tissue.

Subretinal haemorrhage occurred in 62.1% of the patients with nAMD in the CATT study [26]. Depending on the severity of the bleeding, this is associated with a poorer visual outcome [27]. Haemorrhages lead to mechanical compression, ferrotoxicity and impaired nutrition of the neurosensory retina and the RPE, and may cause irreversible damage to vision [28]. It would therefore be desirable to be able to identify patients at risk of massive subretinal bleeding so they can be monitored and treated more intensively. In our study, however, no typical vascular configurations of MNV associated with the occurrence of subretinal haemorrhage were detected. The systemic risk factors so far known to be associated with haemorrhage in nAMD include age, diastolic blood pressure and treatment with anticoagulants and thrombocyte aggregation inhibitors in combination with arterial hypertension [26].

The first limitation to be mentioned of our study is that this is a retrospective study. Another limitation is the manual demarcation of MNV from the surrounding tissue. Moreover, the OCTA technique has inherent limitations: Corneal opacities, pronounced cataract, retinal haemorrhage, the RPE and all vitreous and retinal structures cause shadowing and reflection phenomena and thus artefacts. In addition, OCTA permits optical imaging of the detected blood flow only in a defined time window, and scans with variable interscan time analysis (VISTA) show different vascular structures with different scanning times [29]; therefore, we do not know how closely the vascular structure visualised by OCTA corresponds to reality. Moreover, en-face imaging does not adequately depict a three-dimensional vascular structure, so three-dimensional visualisation and analysis should be a focus of future research. Detailed analyses are also dependent on image quality. While we have excluded patients with reduced image quality, lens status and other media opacities between the OCTA device and the retina can also have an impact on imaging [30].

In conclusion, although the vascular morphology of MNV has several distinctive characteristics, these do not yet have a crucial role in determining disease activity or the best treatment. Currently, the most prominent part in evaluating activity and prognostic factors is played by retinal morphology. OCTA offers the potential for analysing the vascular structure of MNV in detail, rather than just assessing secondary changes. There are thus grounds for optimism that further studies will identify and verify OCTA-based biomarkers, enabling more individualised treatment of nAMD in the future.

## Data Availability

All data used to support the findings of this study are included within the article and are available from the corresponding author upon reasonable request.
